# Formation of dsRNA by-products during *in vitro* transcription can be reduced by using low steady-state levels of UTP

**DOI:** 10.3389/fmolb.2023.1291045

**Published:** 2023-12-11

**Authors:** Thomas Ziegenhals, Ronja Frieling, Philipp Wolf, Katharina Göbel, Stina Koch, Mia Lohmann, Markus Baiersdörfer, Stephanie Fesser, Ugur Sahin, Andreas N. Kuhn

**Affiliations:** BioNTech SE, Mainz, Germany

**Keywords:** mRNA-based therapeutics, *in vitro* transcription, double-stranded RNA, RNA capping, mRNA translatability

## Abstract

**Introduction:** Exogeneous messenger ribonucleic acid (mRNA) can be used as therapeutic and preventive medication. However, during the enzymatic production process, commonly called *in vitro* transcription, by-products occur which can reduce the therapeutic efficacy of mRNA. One such by-product is double-stranded RNA (dsRNA). We therefore sought to limit the generation of dsRNA by-products during *in vitro* transcription.

**Materials and methods:** *In vitro* transcription was performed with a DNA template including a poly(A)-tail-encoding region, dinucleotide or trinucleotide cap analogs for cotranscriptional capping, and relevant nucleoside triphosphates. Concentrations of UTP or modified UTP (m1ΨTP) and GTP were reduced and fed over the course of the reaction. mRNA was analyzed for dsRNA contamination, yield of the reaction, RNA integrity, and capping efficiency before translational activity was assessed.

**Results:** Limiting the steady-state level of UTP or m1ΨTP during the enzymatic reaction reduced dsRNA formation, while not affecting mRNA yield or RNA integrity. Capping efficiency was optimized with the use of a combined GTP and UTP or m1ΨTP feed, while still reducing dsRNA formation. Lower dsRNA levels led to higher protein expression from the corresponding mRNAs.

**Discussion:** Low steady-state concentrations of UTP and GTP, fed in combination over the course of the *in vitro* transcription reaction, produce mRNA with high capping and low levels of dsRNA formation, resulting in high levels of protein expression. This novel approach may render laborious purification steps to remove dsRNA unnecessary.

## 1 Introduction

The pre-clinical and clinical advancement of messenger ribonucleic acid (mRNA)-based therapeutics and vaccines have received a major boost since the successful use of mRNA vaccines in the fight against the COVID-19 pandemic. mRNA-based therapies have huge therapeutic potential in many areas of high unmet medical need, including cancer and infectious diseases, and are under rapid development (Sahin et al., 2014; Sahin et al., 2020; Dousis et al., 2023).

mRNA-based therapies work by translating exogeneous mRNA into the active protein (Wolff et al., 1990). Exogenous mRNA is produced using a cell-free process called *in vitro* transcription, whereby a linear DNA template is transcribed into the corresponding mRNA using a single-subunit phage RNA polymerase (Konarska et al., 1984)—most commonly the RNA polymerase from the T7 phage is used (Chamberlin and Ring, 1973; Cheetham et al., 1999). For efficient translation, and to increase mRNA stability, the mRNA is capped at the 5′ end and a poly(A)-tail is included at the 3′end (Gao et al., 2000; Galloway and Cowling, 2019). For capping, cap analogs can be added to the *in vitro* transcription reaction that are co-transcriptionally incorporated (Konarska et al., 1984; Pasquinelli et al., 1995; Stepinski et al., 2001).

mRNA synthesis using *in vitro* transcription gives rise to by-products that can have unwanted side effects. One of the major by-products of concern is double-stranded RNA (dsRNA) which can cause immunogenic reactions by inducing type I interferons and reduce protein expression (Kariko et al., 2011). Run-off transcription at the 3′ end of the DNA template, which can lead to prolonged RNA extension in the form of “loop back RNA” or “backward transcribed” RNA, is a significant source of dsRNA during *in vitro* transcription (Triana-Alonso et al., 1995; Gholamalipour et al., 2018; Dousis et al., 2023): While dsRNA can be reduced in subsequent purification steps (Kariko et al., 2011; Baiersdorfer et al., 2019), this typically leads to a decrease in mRNA yield and can impact mRNA integrity. Therefore, altering the manufacturing process to reduce formation of dsRNA may be more efficient. This has previously been achieved by increasing the temperature (Wu et al., 2020), adapting the Mg^2+^ concentration (Mu et al., 2018), adding chaotropic agents to the reaction (Piao et al., 2022), or using a mutated T7 RNA polymerase (Dousis et al., 2023).

When using DNA templates that encode a poly(A)-tail, extension of the transcribed RNA in the reverse orientation starts with a poly(A)-tail. Therefore, we hypothesized that reducing the concentration of UTP in the reaction [or a corresponding modified nucleotide, such as N1-methyl-pseudouridine triphosphate (m1ΨTP)] would negatively impact the backward reaction, leading to less dsRNA by-products. Here, we show that limiting UTP via stepwise addition of UTP over the course of the *in vitro* transcription reaction reduces dsRNA formation. Furthermore, combining a UTP and GTP feed reduces dsRNA formation while keeping the capping efficiency high. Importantly, the lower dsRNA levels obtained in this manner led to higher protein expression from the corresponding mRNAs.

## 2 Material and methods

### 2.1 mRNA synthesis

*In vitro* transcription was performed in the presence of a DNA template (linearized plasmid DNA or PCR product) including the coding region for a poly(A)-tail (A_30_L_10_A_70_), either a dinucleotide (m^2,7,3′−O^GppSpG, denoted beta-S-ARCA(D1) (Kuhn et al., 2010) or a trinucleotide (m^2,7,2′−O^Gppp (m^2′−O^)ApG, sold as CleanCap^®^ by TriLink BioTechnologies) cap analog for co-transcriptional capping, and relevant nucleoside triphosphates (GTP, ATP, UTP or m1ΨTP, CTP, Thermo Fisher Scientific) with a final concentration of 9 mM each. The starting concentrations of GTP and/or UTP/m1ΨTP were reduced to 0.5 mM and fed-in 4 or 11 times in equal amounts over the course of the transcription reaction until the final concentration was reached with an additional incubation time of 30 min after the last feed. The transcribed RNAs code for the sequences listed in Supplementary Table S1. The reaction was performed at 37°C with HEPES buffer (40 mM pH 8,3) containing magnesium acetate (40 mM), dithiothreitol (10 mM), and spermidine (2 mM) in the presence of a T7 RNA polymerase, RNase inhibitor (Ribolock, Thermo Fisher Scientific) and inorganic pyrophosphatase (Thermo Fisher Scientific). After *in vitro* transcription, the DNA template was hydrolyzed via DNase I digestion (Thermo Fisher Scientific) and the RNA was purified using magnetic beads for immobilization (Berensmeier, 2006). RNA was eluted in H_2_O. Purified RNA was used for further analysis. Concentration was analyzed by UV spectroscopy and the yield as an indicator for the efficiency of the reaction was calculated in µg RNA per µl of *in vitro* transcription reaction. RNA integrity was analyzed using fragment analyzer (Agilent). Further, the RNA was analyzed for dsRNA contamination and capping efficiency. If indicated, RNA translation was analyzed using a luciferase assay.

### 2.2 Double-stranded (ds)RNA

To determine the amount of dsRNA, 1 µg of RNA was spotted onto a nylon blotting membrane [Nytran SuPerCharge Nylon Blotting Membrane (GE Healthcare Life Sciences, Cat #10416216)] in triplicates, which was then blocked for 1 h in TBS-T buffer containing 5% (w/v) skimmed milk powder. For detection of dsRNA the membrane was incubated for 1 h with J2 dsRNA-specific mouse monoclonal antibody (English and Scientific Consulting, Szirák, Hungary). After washing with TBS-T the membrane was incubated for 1 h with HRP-conjugated donkey anti-mouse IgG (Jackson ImmunoResearch, Cat #715-035-150) washed with TBS-T and developed using Amersham ECL Prime Western Blotting Detection Reagent (Fisher Scientific, Cat # RPN2232) and the ChemiDoc MP Imaging system (Bio-Rad Laboratories, Inc.). Signal intensities were quantified by densitometry using Image Lab 5.1 software (Bio-Rad Laboratories, Inc.). A standard covering the range of 0–1,280 pg dsRNA/µg RNA was part of the dotblot. Densitometric values were evaluated using a standard curve fit in sigma blot.

### 2.3 Capping

To test capping efficiency, the RNA was digested by a P1 3′ exonuclease. The resulting nucleotides were separated with high-performance liquid chromatography and measured by mass spectrometry (Shimadzu Prominence LC-20). Of interest are the 5′-end nucleotides. Capped RNA will leave an NpppN dinucleotide while uncapped RNA will leave a triphosphate-nucleotide (pppN). Capping efficiency was calculated as the ratio of NpppN and the sum of NpppN and pppN.

### 2.4 Cell culture

For generation of human immature DCs (hiDCs), PBMCs were isolated from Buffy Coats by Ficoll gradient and CD14^+^ cells were selected via human CD14 Microbeads (130-050-201, Miltenyi). CD14^+^ cells were incubated for 5 days with 0.2 ng/μL IL4 (130-093-924, Miltenyi) and GM-CSF (130-093-868, Miltenyi). Differentiation into DCs was controlled via FACS with a double CD83 (551073, BD Pharmingen) and CD209 (551265, BD Pharmingen) staining.

### 2.5 Luciferase assay

To prepare lipoplexes for transfection, reporter firefly luciferase (Luc)-encoding RNA was incubated with liposomes following proprietary inhouse protocols. RNA was provided as a HEPES-buffered solution and diluted with 1.5 M NaCl and water to a concentration of 150 mM NaCl and an RNA concentration of 0.1 mg/mL, which results in a molar charge ratio of 0.65:1.00 (positive:negative) upon incubation with liposomes. The charge ratio was calculated from the number of positive charges present in lipid head groups and the number of negative charges derived from the number of RNA nucleotides (330 Da per nucleotide was assumed). Liposomes were in-house generated containing (R)-N,N,N-trimethyl-2-3-dioleyloxy-1-propanammonium chloride (DOTMA) and 1,2-dioleoyl-sn-glycero-3-phosphoethanolamine (DOPE) in a molar ratio of 2:1 (DOTMA:DOPE). Lipoplexes were then mixed with hiDCs, and the transfected cells were seeded in 96-well plates with a density of 5 × 10^4 cells and 0.5 µg RNA per well, in media containing 0.4 ng/μL IL4 and GM-CSF. Luciferase assays were conducted at 6, 24, 48 and 72 h after transfection using the Promega BrightGlo Kit according to manufacturer’s instructions.

## 3 Results

### 3.1 Increasing the cap analog concentration reduces formation of dsRNA

**FIGURE 1:**
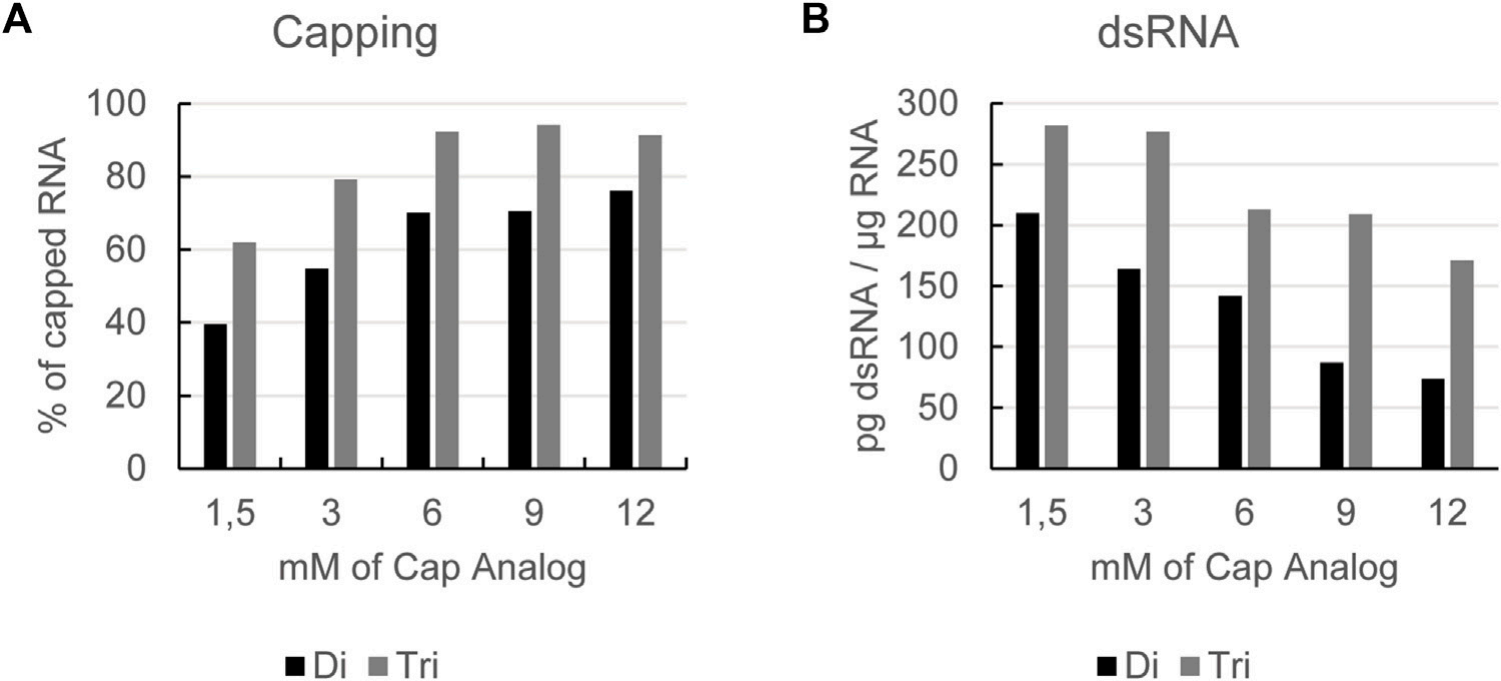
Increasing cap analog concentration leads to higher capping efficiency **(A)** and a reduction in dsRNA formation **(B)**: RNAs produced by *in vitro* transcription with increasing concentrations of either a dinucleotide (Di) or a trinucleotide (Tri) cap analog using a GTP fed-batch reaction were analyzed for capping efficiency **(A)** and dsRNA levels **(B)**. Representative example of several experiments with similar outcomes.

When performing co-transcriptional capping, the cap analog should be kept in excess over the competing nucleoside triphosphate for initiation (most commonly GTP) to obtain a high capping efficiency. To achieve a high yield from the reaction, i.e., to not be limited by a low concentration of GTP, we perform a so-called fed-batch reaction. Here, we start with a low concentration of GTP and then feed in more GTP over the course of the reaction. To optimize capping efficiency of *in vitro* transcribed mRNA, we tested increasing concentrations of cap analogs. This was done with both dinucleotide and trinucleotide cap analogs. As expected, we observed that increasing the cap analog concentration led to higher capping efficiency (Figure 1A). Under these conditions, capping efficiency was higher for the trinucleotide cap analog versus the dinucleotide cap analog (Figure 1A). For both the di- and tri-nucleotide cap analogs, a plateau was reached at a concentration of 6 mM with respect to the capping degree of the obtained mRNA.

Further analysis of the mRNA showed that cap concentration had no impact on reaction yield or RNA integrity (Supplementary Table S1). However, when analyzing the dsRNA level, we observed that by increasing either cap analog concentration, the formation of dsRNA was reduced (Figure 1B). This was the case for cap concentrations of up to 12 mM, i.e., beyond the levels shown to have an impact on the capping efficiency of *in vitro* transcribed mRNA.

### 3.2 *In vitro* transcription with a UTP feed lowers dsRNA formation in poly(A)-carrying transcripts

For poly(A)-encoding DNA templates, where backwards transcription at the 3′ end of the DNA template would start with a poly(U)-stretch, we reasoned that a lower concentration of UTP might limit the number of backward reactions, leading to lower dsRNA levels.

**FIGURE 2:**
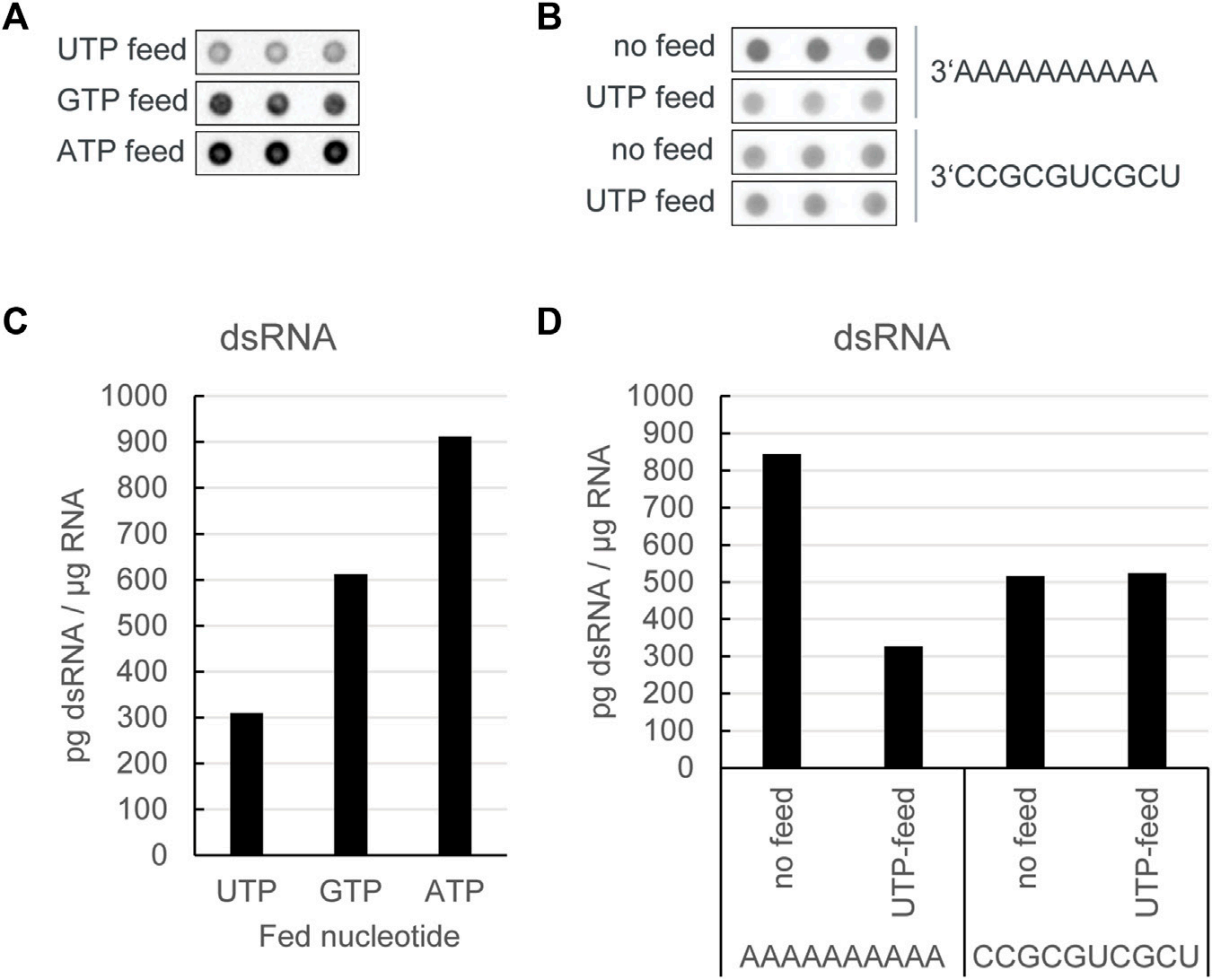
*In vitro* transcription reactions with a UTP feed resulted in production of mRNA with the lowest level of dsRNA. **(A)** J2 dotblot of *in vitro* transcription reactions with a dinucleotide cap analog and a DNA template encoding a poly(A)-tail at the 3′ end, in which different nucleotide triphosphates as indicated were fed over time: **(B)** J2 dotblot of dsRNA levels from *in vitro* transcription RNA from DNA templates encoding different 3′ ends either with no nucleotide triphosphate feed or with a UTP feed: Quantification of dsRNA levels as shown in **(C, D)**. Representative example of several experiments with similar outcomes.

To test this hypothesis, we performed an *in vitro* transcription of an unmodified RNA using the dinucleotide cap analog reaction with a low starting concentration of UTP. To reduce the impact on the reaction yield, we then fed UTP into the reaction over time at a rate similar to the GTP feed. Reactions with a GTP feed or an ATP feed were used as controls. Overall, *in vitro* transcription reactions with a UTP feed resulted in production of mRNA with the lowest level of dsRNA, while limitation of ATP was shown to increase dsRNA formation versus the GTP feed as control (Figure 2A and Figure 2C). Notably, the reduction in dsRNA was only seen if the DNA template encoded a poly(A)-tail at the 3′ end, and not with templates ending with a more random nucleotide distribution (Figure 2B and Figure 2D), thereby corroborating our hypothesis that limiting UTP reduces the occurrence of a backward reaction, thus giving rise to lower levels of dsRNA by-products.

### 3.3 A combined feed of GTP and UTP reduces dsRNA formation while keeping capping efficiency high

**FIGURE 3:**
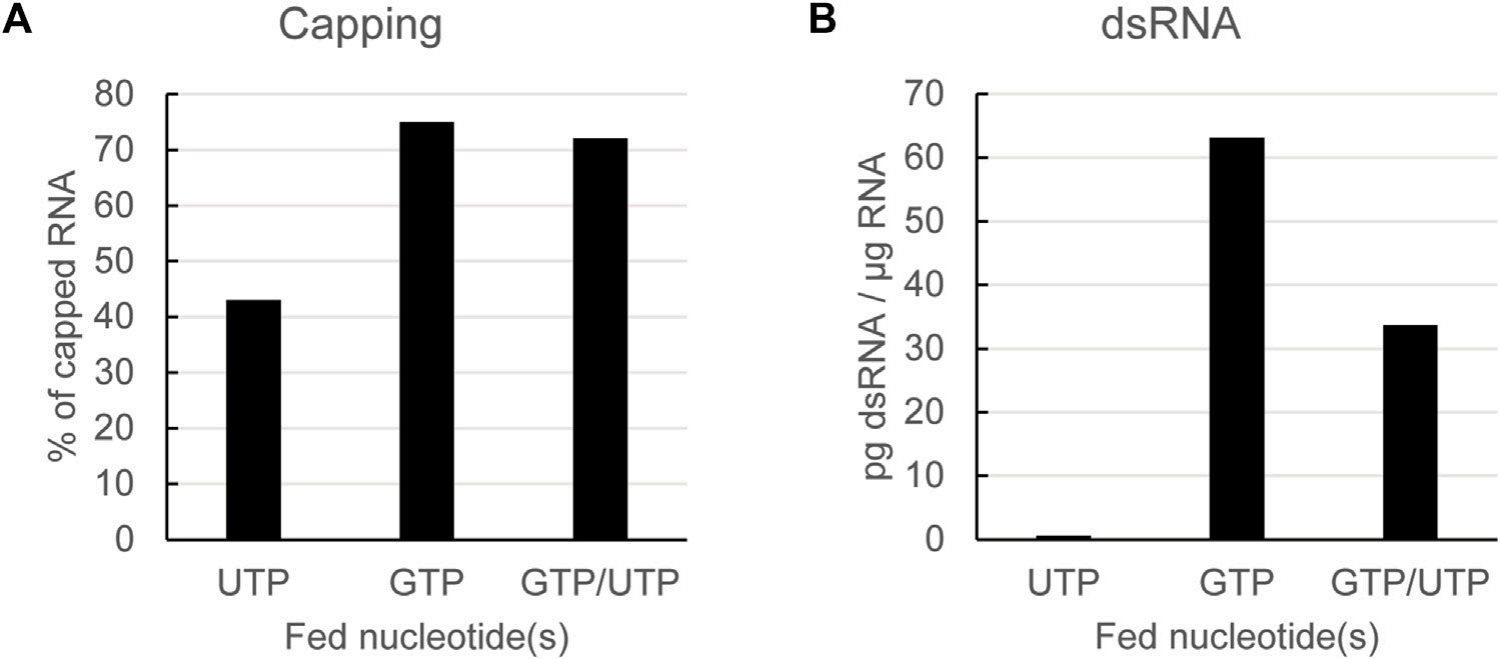
Using a combined GTP and UTP feed keeps capping efficiency high while still reducing dsRNA formation. Analysis of *in vitro* transcribed RNA with a dinucleotide cap analog fed with the indicated nucleotides. Capping efficiency is reduced using a UTP feed, but can be rescued to GTP feed levels with a combined GTP/UTP feed **(A)**: dsRNA is reduced using UTP feed or GPT/UTP dual feed **(B)**: Representative example of several experiments with similar outcomes.

As already shown, lowering the steady-state concentration of UTP during *in vitro* transcription leads to lower levels of dsRNA formation. However, as the GTP concentration is not lowered, as expected, capping is suboptimal in reactions applying a UTP feed with dinucleotide co-transcriptional capping (Figure 3A). We subsequently tested whether we could optimize capping while still reducing dsRNA formation by using a combined feed of GTP and UTP. The capping of mRNAs obtained from a combined GTP/UTP feed was similar to that from a GTP feed alone (Figure 3A), thus indicating that the lower UTP concentration had no negative impact on capping. Additionally, the GTP/UTP feed also lowered formation of dsRNA, albeit not as much as the UTP feed alone (Figure 3B).

For the experiments shown in Figures 2, 3, we used as indicated a dinucleotide cap analog and non-modified UTP. To check if the positive effect of lower steady-state levels of UTP, alone or in combination with GTP, on dsRNA levels also held true for trinucleotide cap analogs and/or modified UTPs, we performed a set of reactions in which we used all four combinations: a dinucleotide or a trinucleotide cap analog with either UTP or N1-methyl-pseudouridine triphosphate (m1ΨTP). In each case, we fed either GTP, UTP or m1ΨTP alone, or GTP together with either UTP or m1ΨTP, and analyzed capping efficiency and dsRNA levels of the resulting mRNAs. For the reactions with a GTP feed, reaction conditions were adapted to give similar levels of capping for both the di- and tri-nucleotide cap analogs. In this set of experiments, the RNAs were coding for luciferase, which allowed to also test their translatability (see below).

**FIGURE 4:**
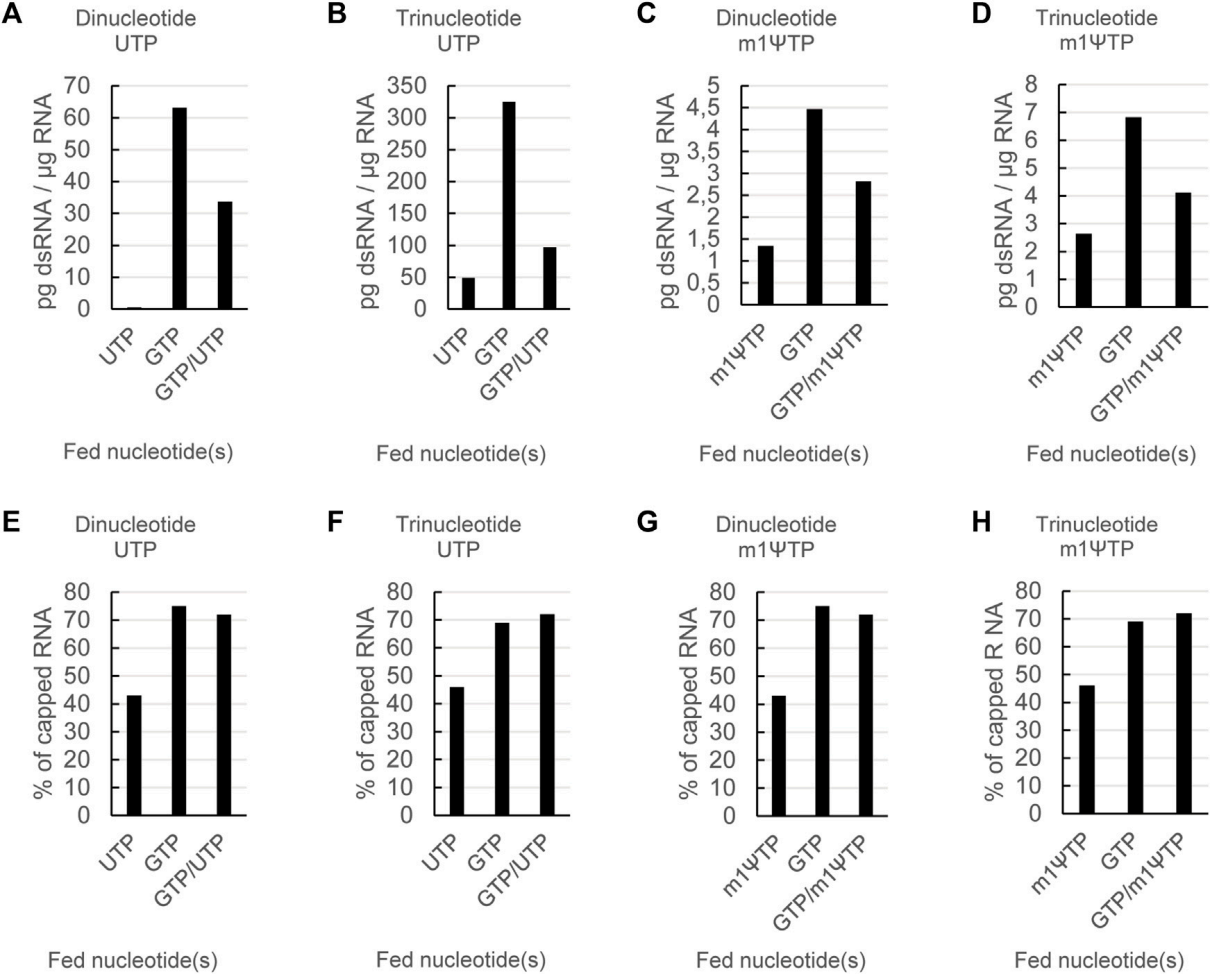
The effects of nucleotide feeds on dsRNA and capping are independent of the type of cap analog and whether modified or unmodified UTP is used. RNAs were *in vitro* transcribed either in the presence of a dinucleotide or a trinucleotide cap analog and either using UTP or m1ΨTP with feeding of the indicated nucleotide triphosphates. The resulting RNAs were then analyzed for dsRNA levels **(A)** and capping **(B)**. Please note the different scales for the dsRNA levels in **(A)**.

Lowering the steady-state concentration of UTP or m1ΨTP led to a reduction of dsRNA formation for all four combinations (Figures 4A–D). Similarly, capping efficiency was not affected by feeding either UTP or m1ΨTP in combination with GTP, as capping levels obtained with combined feeds were similar to levels obtained with GTP-only feeds (Figures 4E–H). As already shown for a dinucleotide cap with UTP (Figure 3), in all four combinations combined feeds reduced levels of dsRNA to a lesser extent than single feeds with only UTP or m1ΨTP (Figures 4A–D). Lastly, we observed that dsRNA levels were higher for the reactions with a trinucleotide cap analog. This is more pronounced for reactions with UTP than m1ΨTP. In summary, optimized mRNA production—with high capping and low levels of dsRNA—can be obtained by using a combined feed of GTP and (modified) UTP, independent of the type of cap analog.

To further demonstrate the advantages of the dual feed, we performed the same experiment with RNAs coding for eGFP (Supplementary Figure S1). The effect of the different nucleotide feeds as seen for the luciferase RNA could also be observed for the RNAs encoding eGFP, showing that this is a general effect independent of the mRNA sequence.

### 3.4 Translational activity is increased for mRNAs produced with a combined feed of GTP and UTP

For mRNAs with a high capping efficiency and low levels of dsRNA, high protein expression was expected because: (i) more mRNAs would be expected to be translated and (ii) less type I interferons should be induced, therefore reducing repression of translation. To verify this, we analyzed the same set of mRNAs coding for luciferase as described above for translatability in cell culture.

**FIGURE 5:**
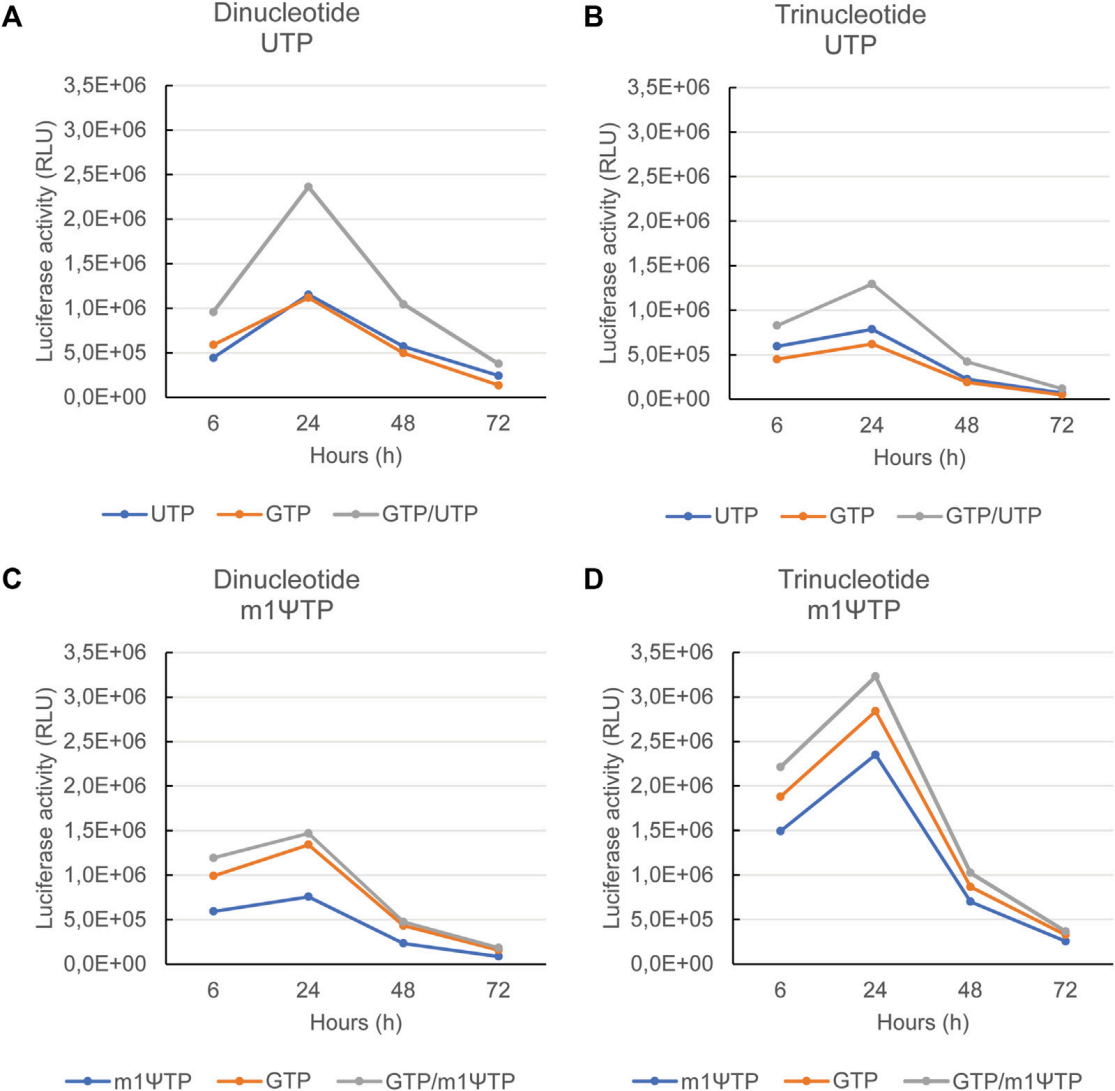
Maximal protein expression is observed with mRNAs transcribed using a combined feed of GTP and UTP or m1ΨTP. The mRNAs as also used for the experiments in Figure 4, all coding for luciferase, were transfected into human immature dendritic cells. Upon transfection, luciferase activity was measured 6, 24, 48, and 72 h after transfection. The luciferase activity is depicted for mRNAs capped with a dinucleotide cap analog and using UTP in **(A)**, capped with a trinucleotide cap analog and using UTP in **(B)**, capped with a dinucleotide cap analog and using m1ΨTP in **(C)**, and capped with a trinucleotide cap analog and using m1ΨTP in **(D)**. In each panel, the corresponding feeds are indicated at the bottom. Representative example of several experiments with similar outcomes.

For each of the four combinations (di- or tri-nucleotide, with UTP or m1ΨTP), maximal protein expression was observed for the mRNAs produced with the combined feed, GTP and UTP or m1ΨTP (Figure 5). This demonstrated—as expected—that high capping efficiency and low levels of dsRNA increase the translational activity of mRNAs. However, some differences were still observed between the four combinations. For unmodified mRNAs using a dinucleotide cap analog, the single feeds of GTP or UTP yielded about the same level of protein expression (Figure 5A). This suggests that the higher capping with the GTP feed has a similar impact on translation as the lower dsRNA amount with the UTP feed.

As previously shown the combination of unmodified RNA with a trinucleotide cap analog yielded the highest levels of dsRNA (Figure 4A). This might be the reason why with each of the three feeds, protein expression is lowest for the mRNAs capped with a trinucleotide versus those capped with a dinucleotide cap analog (Figures 5A, B). With the trinucleotide cap analog, the UTP feed alone yielded slightly higher protein expression compared with the GTP feed alone (Figure 5C). For the modified mRNAs, i.e., transcribed using m1ΨTP, the dsRNA levels were low for the mRNAs capped with either a di- or a tri-nucleotide cap structure, even with the GTP feed. However, protein expression was still higher for mRNAs transcribed with a combined GTP and m1ΨTP feed. Interestingly, luciferase activity for an mRNA produced with a m1ΨTP feed compared with a combined feed is higher with a dinucleotide cap structure (Figures 5C, D), which may be due to differences in cap structure, as the capping efficiencies for the pairs of mRNAs with the same feed are approximately equal.

Overall, our data show that formation of dsRNA by-products during *in vitro* transcription can be reduced for mRNAs with a poly(A)-tail at the 3′ end by keeping the steady-state concentration of UTP (or m1ΨTP) low. When this is combined with a GTP feed for high capping efficiency, the corresponding mRNAs exhibit increased protein expression in cell culture, in line with the expected effect of highly capped mRNA containing less dsRNA.

## 4 Discussion

mRNAs used as therapeutics or vaccines need to be translated with high efficiency, while at the same time inducing low reactogenicity. Therefore, reducing production of by-products, that can cause immunogenic reactions, such as dsRNA, while retaining translational efficiency is desirable (Kariko et al., 2011). In this study we found that increasing the di- or tri-nucleotide cap analog concentration reduced dsRNA formation and limiting UTP over the course of the *in vitro* transcription reaction led to reduced dsRNA formation in poly(A)-carrying transcripts. Additionally, by combining UTP and GTP feeds, high capping efficiency with reduced dsRNA formation was achieved. Lower dsRNA levels led to higher protein expression from the corresponding mRNAs.

Mechanistically, it is difficult to reason why increasing cap analog concentration results in reduced formation of dsRNA. One possible explanation is that initiation of the reaction is more efficient because higher amounts of cap analog leads to more free promotor sequences, which are available to be bound by T7 RNA polymerase. This in turn might favor the release of T7 RNA polymerases from the DNA templates upon reaching the 3′ end, as all these processes would be expected to happen in equilibrium to a certain extent.

As previously mentioned, during *in vitro* transcription, a major source of dsRNA formation is backward transcription once the RNA polymerase reaches the 3′ end of the DNA template rather than termination (Gholamalipour et al., 2018; Dousis et al., 2023). For DNA templates with an encoded poly(A)-tail these backward transcription events start with a poly(U)-stretch. Here we showed that starting *in vitro* transcription with a low concentration of UTP or m1ΨTP, and then feeding additional UTP or m1ΨTP over the course of the reaction, reduced levels of dsRNA independent of the type of cap analog and regardless of whether UTP or m1ΨTP was used. As expected, this effect was specific for DNA templates encoding a poly(A)-tail at the 3′ end.

Reaching a satisfactory capping efficiency with co-transcriptional capping is a challenge because the cap analog competes with the starting nucleotide for the initiation of a new mRNA. T7 RNA polymerase favors starting transcription with a G (Chamberlin and Ring, 1973) therefore GTP is used at a lower concentration and then fed to the reaction when using dinucleotide cap analogs. As T7 RNA polymerase also has a tendency to skip the first base when transcribing, it is still recommended to have the ratio of cap to GTP as high as possible during the reaction also for the use of AG starting trinucleotides. However, cap analogs are expensive and therefore reducing the GTP concentration is a less expensive way to achieve a ratio in favor of the cap analog rather than further increasing the concentration of the cap analog, with GTP being replenished over the course of the reaction via a GTP feed. Importantly, we observed a reduction in dsRNA formation when UTP and GTP feeds were combined to ensure a high capping efficiency. However, dsRNA reduction was not as pronounced with the combined feed versus the singular UTP or m1ΨTP feeds. This may be because the low steady-state concentration of GTP leads to impaired transcription initiation. Consequently, this may have partially counteracted the effect of using lower steady-state levels of UTP or m1ΨTP on dsRNA formation. Capping efficiency was improved with both dinucleotide and trinucleotide analogs. Capping efficiency is further improved by keeping the GTP concentration low and this can save costs with respect to expensive cap analogs.

Testing mRNAs produced with different nucleoside triphosphate feeds also allowed us to gain some insight into how capping and dsRNA formation impact protein expression, which may be informative for both pre-clinical and clinical research. Importantly, we showed that mRNAs produced using a combined feed gave the highest levels of protein expression versus singular feeds. This indicates that reducing dsRNA formation during *in vitro* transcription, by lowering the steady-state concentration of UTP, increases protein expression. The impact of reducing dsRNA levels on protein expression is more pronounced for mRNA transcribed with UTP (non-modified mRNA) compared with mRNA transcribed with m1ΨTP (modified mRNA), which is reflected in the absolute amount of dsRNA.

While higher levels of capping appear to correlate with higher levels of protein expression, the correlation may be weaker with trinucleotide versus dinucleotide cap analogs. However, it is difficult to determine whether this may be due to differences between the cap analogs themselves or the effect of different cap structures, i.e., cap0 versus cap1. High capping and low levels of dsRNA formation led to increased translational activity for non-modified and modified mRNAs transcribed in the presence of dinucleotide or trinucleotide cap analogs. The potential benefit of a cap1 structure (Kumar et al., 2014), as obtained with the trinucleotide cap analog, is apparently counteracted by the higher amounts of dsRNA observed with trinucleotide cap analogs compared with dinucleotide analogs, as the corresponding mRNAs all showed lower protein expression. However, it remains unknown why trinucleotide cap analogs lead to higher levels of dsRNA. Notably, the capping assay we used cannot differentiate between capped mRNA species that have aberrantly initiated and nucleotides that have been added or missed by T7 RNA polymerase. Thus, further work—which is beyond the scope of this study—will be needed to better understand the effects of different cap structures and dsRNA formation on mRNA in reactions with different nucleotide triphosphate feeds.

With the increasing interest in mRNA-based therapies, improved manufacturing processes are needed to allow this technology to reach its full potential. *In vitro* transcription reactions should be optimized when synthesizing exogenous mRNAs. Here, we have shown that low steady-state concentrations of UTP and GTP, fed in combination over the course of the *in vitro* transcription reaction, produces mRNA with high capping and low levels of dsRNA formation, resulting in high levels of protein expression. This novel approach may render laborious purification steps to remove dsRNA unnecessary.

## Data Availability

The raw data supporting the conclusion of this article will be made available by the authors, without undue reservation.

## References

[B1] Baiersdorfer M. Boros G. Muramatsu H. Mahiny A. Vlatkovic I. Sahin U. (2019). A facile method for the removal of dsRNA contaminant from *in vitro*-Transcribed mRNA. Mol. Ther. Nucleic Acids 15, 26–35. 10.1016/j.omtn.2019.02.018 30933724 PMC6444222

[B2] Berensmeier S. (2006). Magnetic particles for the separation and purification of nucleic acids. Appl. Microbiol. Biotechnol. 73 (3), 495–504. 10.1007/s00253-006-0675-0 17063328 PMC7080036

[B3] Chamberlin M. Ring J. (1973). Characterization of T7-specific ribonucleic acid polymerase. J. Biol. Chem. 248 (6), 2235–2244. 10.1016/S0021-9258(19)44211-7 4570474

[B4] Cheetham G. M. T. Jeruzalmi D. Steitz T. A. (1999). Structural basis for initiation of transcription from an RNA polymerase–promoter complex. Nature 399 (6731), 80–83. 10.1038/19999 10331394

[B5] Dousis A. Ravichandran K. Hobert E. M. Moore M. J. Rabideau A. E. (2023). An engineered T7 RNA polymerase that produces mRNA free of immunostimulatory byproducts. Nat. Biotechnol. 41 (4), 560–568. 10.1038/s41587-022-01525-6 36357718 PMC10110463

[B6] Galloway A. Cowling V. H. (2019). mRNA cap regulation in mammalian cell function and fate. Biochim. Biophys. Acta Gene Regul. Mech. 1862 (3), 270–279. 10.1016/j.bbagrm.2018.09.011 30312682 PMC6414751

[B7] Gao M. Fritz D. T. Ford L. P. Wilusz J. (2000). Interaction between a poly(A)-specific ribonuclease and the 5' cap influences mRNA deadenylation rates *in vitro* . Mol. Cell 5 (3), 479–488. 10.1016/s1097-2765(00)80442-6 10882133 PMC2811581

[B8] Gholamalipour Y. Karunanayake Mudiyanselage A. Martin C. T. (2018). 3' end additions by T7 RNA polymerase are RNA self-templated, distributive and diverse in character-RNA-Seq analyses. Nucleic Acids Res. 46 (18), 9253–9263. 10.1093/nar/gky796 30219859 PMC6182178

[B9] Kariko K. Muramatsu H. Ludwig J. Weissman D. (2011). Generating the optimal mRNA for therapy: HPLC purification eliminates immune activation and improves translation of nucleoside-modified, protein-encoding mRNA. Nucleic Acids Res. 39 (21), e142. 10.1093/nar/gkr695 21890902 PMC3241667

[B10] Konarska M. M. Padgett R. A. Sharp P. A. (1984). Recognition of cap structure in splicing *in vitro* of mRNA precursors. Cell 38 (3), 731–736. 10.1016/0092-8674(84)90268-X 6567484

[B11] Kuhn A. N. Diken M. Kreiter S. Selmi A. Kowalska J. Jemielity J. (2010). Phosphorothioate cap analogs increase stability and translational efficiency of RNA vaccines in immature dendritic cells and induce superior immune responses *in vivo* . Gene Ther. 17 (8), 961–971. 10.1038/gt.2010.52 20410931

[B12] Kumar P. Sweeney T. R. Skabkin M. A. Skabkina O. V. Hellen C. U. Pestova T. V. (2014). Inhibition of translation by IFIT family members is determined by their ability to interact selectively with the 5'-terminal regions of cap0-cap1- and 5'ppp-mRNAs. Nucleic Acids Res. 42 (5), 3228–3245. 10.1093/nar/gkt1321 24371270 PMC3950709

[B13] Mu X. Greenwald E. Ahmad S. Hur S. (2018). An origin of the immunogenicity of *in vitro* transcribed RNA. Nucleic Acids Res. 46 (10), 5239–5249. 10.1093/nar/gky177 29534222 PMC6007322

[B14] Pasquinelli A. E. Dahlberg J. E. Lund E. (1995). Reverse 5' caps in RNAs made *in vitro* by phage RNA polymerases. RNA 1 (9), 957–967. 8548660 PMC1369344

[B15] Piao X. Yadav V. Wang E. Chang W. Tau L. Lindenmuth B. E. (2022). Double-stranded RNA reduction by chaotropic agents during *in vitro* transcription of messenger RNA. Mol. Ther. Nucleic Acids 29, 618–624. 10.1016/j.omtn.2022.08.001 36090758 PMC9421179

[B16] Sahin U. Kariko K. Tureci O. (2014). mRNA-based therapeutics--developing a new class of drugs. Nat. Rev. Drug Discov. 13 (10), 759–780. 10.1038/nrd4278 25233993

[B17] Sahin U. Muik A. Derhovanessian E. Vogler I. Kranz L. M. Vormehr M. (2020). COVID-19 vaccine BNT162b1 elicits human antibody and T(H)1 T cell responses. Nature 586 (7830), 594–599. 10.1038/s41586-020-2814-7 32998157

[B18] Stepinski J. Waddell C. Stolarski R. Darzynkiewicz E. Rhoads R. E. (2001). Synthesis and properties of mRNAs containing the novel "anti-reverse" cap analogs 7-methyl(3'-O-methyl)GpppG and 7-methyl (3'-deoxy)GpppG. RNA 7 (10), 1486–1495. 11680853 PMC1370192

[B19] Triana-Alonso F. J. Dabrowski M. Wadzack J. Nierhaus K. H. (1995). Self-coded 3'-extension of run-off transcripts produces aberrant products during *in vitro* transcription with T7 RNA polymerase. J. Biol. Chem. 270 (11), 6298–6307. 10.1074/jbc.270.11.6298 7534310

[B20] Wolff J. A. Malone R. W. Williams P. Chong W. Acsadi G. Jani A. (1990). Direct gene transfer into mouse muscle *in vivo* . Science 247 (4949 Pt 1), 1465–1468. 10.1126/science.1690918 1690918

[B21] Wu M. Z. Asahara H. Tzertzinis G. Roy B. (2020). Synthesis of low immunogenicity RNA with high-temperature *in vitro* transcription. RNA 26 (3), 345–360. 10.1261/rna.073858.119 31900329 PMC7025508

