# Supplementary material for: Formation of dsRNA by-products during in vitro transcription can be reduced by using low steady-state levels of UTP
Source: Front Mol Biosci. 2023 Dec 11;10:1291045. doi: 10.3389/fmolb.2023.1291045 (PMC10749352; doi:10.3389/fmolb.2023.1291045)
Supplement: Supplementary file 1 [file DataSheet1.docx]

Supplementary Material

Formation of dsRNA by-products during *in vitro* transcription can be reduced by using low steady-state levels of UTP

Thomas Ziegenhals*^1^, Ronja Frieling^1^, Philipp Wolf^1^, Katharina Göbel^1^, Stina Koch^1^, Mia Lohmann^1^, Markus Baiersdörfer^1^, Stephanie Fesser^1^, Ugur Sahin^1^, Andreas N. Kuhn^1^

^1^BioNTech SE, Mainz, Germany

*** Correspondence:**Corresponding Author
Thomas.ziegenhals@biontech.de

## Supplementary Table

| **Name** | **ORF** | **Cap Analog** | **RNA length** | **Fed Nucleotide** | **Yield µg RNA / µl IVT** | **Integrity in %** | **Capping Efficiency in %** | **pg dsRNA / µg RNA** | **Figure** |
| --- | --- | --- | --- | --- | --- | --- | --- | --- | --- |
| RNA1 | Luciferase | Dinucleotide | 2135 | GTP | 7,99 | 93 | 40 | 210 | 1 |
| RNA2 | Luciferase | Dinucleotide | 2135 | GTP | 6,92 | 94 | 55 | 164 | 1 |
| RNA3 | Luciferase | Dinucleotide | 2135 | GTP | 7,79 | 94 | 70 | 142 | 1 |
| RNA4 | Luciferase | Dinucleotide | 2135 | GTP | 7,76 | 94 | 71 | 87 | 1 |
| RNA5 | Luciferase | Dinucleotide | 2135 | GTP | 7,89 | 93 | 76 | 74 | 1 |
|  |  |  |  |  |  |  |  |  |  |
| RNA6 | Luciferase | Trinucleottide | 2135 | GTP | 6,59 | 92 | 62 | 282 | 1 |
| RNA7 | Luciferase | Trinucleottide | 2135 | GTP | 6,78 | 93 | 79 | 277 | 1 |
| RNA8 | Luciferase | Trinucleottide | 2135 | GTP | 7,82 | 94 | 92 | 213 | 1 |
| RNA9 | Luciferase | Trinucleottide | 2135 | GTP | 6,29 | 94 | 94 | 209 | 1 |
| RNA10 | Luciferase | Trinucleottide | 2135 | GTP | 7,23 | 94 | 91 | 171 | 1 |
|  |  |  |  |  |  |  |  |  |  |
| RNA11 | Luciferase | Trinucleottide | 2135 | UTP | 7,27 | 86 | 61 | 310 | 2 |
| RNA12 | Luciferase | Trinucleottide | 2135 | GTP | 7,11 | 85 | 82 | 612 | 2 |
| RNA13 | Luciferase | Trinucleottide | 2135 | ATP | 7,17 | 87 | 72 | 913 | 2 |
|  |  |  |  |  |  |  |  |  |  |
| RAN14 | Antigene Cassette | Dinucleotide | 1851 | no Feed | 4,62 | n.a. | n.a. | 844 | 2 |
| RAN15 | Antigene Cassette | Dinucleotide | 1851 | UTP | 4,42 | n.a. | n.a. | 326 | 2 |
| RAN16 | Antigene Cassette | Dinucleotide | 1851 | no Feed | 4,17 | n.a. | n.a. | 516 | 2 |
| RAN17 | Antigene Cassette | Dinucleotide | 1851 | UTP | 4,18 | n.a. | n.a. | 524 | 2 |
|  |  |  |  |  |  |  |  |  |  |
| RNA18 | Luciferase | Dinucleotide | 2135 | UTP | 9,21 | 86 | 43 | 1 | 3/4/5 |
| RNA19 | Luciferase | Dinucleotide | 2135 | GTP | 9,58 | 94 | 75 | 63 | 3/4/5 |
| RNA20 | Luciferase | Dinucleotide | 2135 | GTP/UTP | 9,26 | 92 | 72 | 34 | 3/4/5 |
| RNA24 | Luciferase | Trinucleottide | 2135 | UTP | 8,84 | 83 | 43 | 1 | 4/5 |
| RNA25 | Luciferase | Trinucleottide | 2135 | GTP | 8,47 | 94 | 71 | 4 | 4/5 |
| RNA26 | Luciferase | Trinucleottide | 2135 | GTP/UTP | 8,05 | 92 | 69 | 3 | 4/5 |
| RNA27 | Luciferase | Dinucleotide | 2135 | m1ΨTP | 9,35 | 91 | 46 | 49 | 4/5 |
| RNA28 | Luciferase | Dinucleotide | 2135 | GTP | 9,18 | 95 | 69 | 325 | 4/5 |
| RNA29 | Luciferase | Dinucleotide | 2135 | GTP/m1ΨTP | 8,57 | 94 | 72 | 97 | 4/5 |
| RNA30 | Luciferase | Trinucleottide | 2135 | m1ΨTP | 7,43 | 91 | 48 | 3 | 4/5 |
| RNA31 | Luciferase | Trinucleottide | 2135 | GTP | 7,82 | 95 | 73 | 7 | 4/5 |
| RNA32 | Luciferase | Trinucleottide | 2135 | GTP/m1ΨTP | 7,79 | 93 | 75 | 4 | 4/5 |
|  |  |  |  |  |  |  |  |  |  |
| RNA33 | eGFP | Dinucleotide | 1202 | UTP | 9,31 | 95 | 41 | 47 | Supp 1 |
| RNA34 | eGFP | Dinucleotide | 1202 | GTP | 8,83 | 97 | 65 | 350 | Supp 1 |
| RNA35 | eGFP | Dinucleotide | 1202 | GTP/UTP | 8,46 | 96 | 70 | 202 | Supp 1 |
| RNA36 | eGFP | Trinucleottide | 1202 | UTP | 7,50 | 97 | 63 | 415 | Supp 1 |
| RNA37 | eGFP | Trinucleottide | 1202 | GTP | 7,69 | 98 | 71 | 666 | Supp 1 |
| RNA38 | eGFP | Trinucleottide | 1202 | GTP/UTP | 7,74 | 96 | 74 | 549 | Supp 1 |
| RNA39 | eGFP | Dinucleotide | 1202 | m1ΨTP | 8,29 | 92 | 40 | 4 | Supp 1 |
| RNA40 | eGFP | Dinucleotide | 1202 | GTP | 8,59 | 97 | 57 | 10 | Supp 1 |
| RNA41 | eGFP | Dinucleotide | 1202 | GTP/m1ΨTP | 7,38 | 95 | 65 | 5 | Supp 1 |
| RNA42 | eGFP | Trinucleottide | 1202 | m1ΨTP | 7,51 | 95 | 66 | 18 | Supp 1 |
| RNA43 | eGFP | Trinucleottide | 1202 | GTP | 6,79 | 97 | 72 | 73 | Supp 1 |
| RNA44 | eGFP | Trinucleottide | 1202 | GTP/m1ΨTP | 6,76 | 96 | 77 | 13 | Supp 1 |

**Table 1** RNA properties of shown RNAs

## Supplementary Figures

**B**

**A**

**Supplementary Figure 1.** eGFP coding RNAs were *in vitro* transcribed either in the presence of a dinucleotide or a trinucleotide cap analog and either using UTP or m1ΨTP with feeding of the indicated nucleotide triphosphates. The resulting RNAs were then analyzed for dsRNA levels (**A**) and capping (**B**). Please note the different scales for the dsRNA levels in (**A**).
